# T cell Tolerance in Early Life

**DOI:** 10.3389/fimmu.2020.576261

**Published:** 2020-11-20

**Authors:** Lijun Yang, Rong Jin, Dan Lu, Qing Ge

**Affiliations:** ^1^ Department of Immunology, School of Basic Medical Sciences, Peking University, NHC Key Laboratory of Medical Immunology (Peking University), Beijing, China; ^2^ Institute of Systems Biomedicine, Peking University Health Science Center, Beijing, China; ^3^ Department of Integration of Chinese and Western Medicine, School of Basic Medical Sciences, Peking University, Beijing, China

**Keywords:** neonatal period, T cell tolerance, regulatory T cells, conventional T cells, allogeneic hematopoietic stem cell transplantation

## Abstract

T cell-mediated immune tolerance is a state of unresponsiveness of T cells towards specific self or non-self antigens. This is particularly essential during prenatal/neonatal period when T cells are exposed to dramatically changing environment and required to avoid rejection of maternal antigens, limit autoimmune responses, tolerate inert environmental and food antigens and antigens from non-harmful commensal microorganisms, promote maturation of mucosal barrier function, yet mount an appropriate response to pathogenic microorganisms. The cell-intrinsic and cell extrinsic mechanisms promote the generation of prenatal/neonatal T cells with distinct features to meet the complex and dynamic need of tolerance during this period. Reduced exposure or impaired tolerance in early life may have significant impact on allergic or autoimmune diseases in adult life. The uniqueness of conventional and regulatory T cells in human umbilical cord blood (UCB) may also provide certain advantages in UCB transplantation for hematological disorders.

## Introduction

Immune tolerance is a state of unresponsiveness of the immune cells towards specific self or non-self antigens. It is an essential mechanism to prevent unwanted or self-reactive immune responses. In allogeneic hematopoietic stem cell transplantation (HSCT), failure to develop immune tolerance to autoantigens and alloantigens results in chronic graft-*versus*-host disease (GVHD), a leading cause of non-relapse morbidity and mortality (1).

Immune tolerance was first discovered in neonatal dizygotic cattle twins with cellular chimerism that was due to naturally occurring placental anastomoses and exchange of non-self antigens (2). Anderson et al. then showed that skin grafts between these calves were well accepted (3). Since then, the concepts of neonatal immune tolerance and transplant tolerance were first described (4, 5).

T cells play an essential role in neonatal immune tolerance. Thymectomy at day 3 (d3Tx) after birth quickly leads to the development of an autoimmune wasting disease in mice which could be rescued by a thymus transplant (6, 7). At the neonatal period (from birth through the first month of life in human or the first 1–2 weeks in mice), T cells are exposed to a rapidly and dramatically changing environment, not only from the thymus to peripheral tissues with variable maturity, but also from a relatively pathogen-free and stable environment *in utero* to the diverse microbial environment in the outside world. During this period, T cells need to avoid rejection of the maternal host, limit autoimmune responses, tolerate inert environmental and food antigens and antigens from non-harmful commensal microorganisms, promote maturation of mucosal barrier function, yet mount an appropriate response to pathogenic microorganisms (8). The clonal deletion of autoreactive T cells in the thymus (central tolerance) (9, 10) and the suppressive activity of regulatory T cells (Tregs) in the periphery (peripheral tolerance) (11–15) are both crucial in immune tolerance. But the mechanisms underlying the uniqueness of neonatal T cell tolerance and its adaptation to the adult state are just beginning to be understood after decades of comparison between neonatal and adult T cells. In this review, we will summarize current knowledge on T cell tolerance in early life and subsequent advantages of umbilical cord blood (UCB) T cells in tolerance development in allogeneic HSCT.

## T Cell Repertoire Before Thymic Selection in Early Life

The stepwise T cell development, selection, and the generation of a functional T cell repertoire occur in the thymus (16). Compared to adult T cells, both human and murine neonatal conventional T (Tconv) cells and Treg cells have shorter T cell receptor (TCR) or shorter complementarity determining region (CDR)3*α* stretches, fewer N-region additions (more germ line-encoded clonotypes), and are less clonally expanded (17–27). Human UCB T cells also revealed higher percentage of nonfunctional TCR*β* mRNAs, likely due to suppressed nonsense-mediated decay mechanism (26). The shorter TCRs in neonatal T cells do not limit TCR diversity. The results from deep sequencing and single cell sequencing demonstrate higher diversity of TCR repertoire in human neonatal Tconv and Tregs when compared to adult ones (28, 29). In addition, UCB Treg cells are also shown to have more clones with TCRs specific for autoantigens (28).

Terminal deoxynuceotidyl transferase (TdT) is responsible for template-independent nucleotide addition during the V(D)J rearrangement. It contributes to 90% of TCR*αβ* diversity. The activity of TdT is believed to be low in the fetal period of both humans and mice. In particular, TdT expression could be only detected until 4–5 days after birth in mice and beyond 20^th^ week of gestation in human. Such delayed TdT expression not only makes a significant contribution to short CDR3 length and less N-addition in TCRs of human and murine neonatal T cells (26, 30–32), but also leads to relatively high numbers of public clonotypes shared among human UCB samples (26).

In addition to different diversity, neonatal TCR repertoire is also biased toward TCRs with high affinity and high cross-reactivity. This is mainly based on the studies of *Tdt*-deficient mice but is confirmed later with other mouse models. T cells lacking *Tdt* showed increased affinity of TCR to the *α* helices of self-MHC (major histocompatibility complex) (33, 34). One of the surface markers that can report the TCR avidity for peptide/MHC complexes is CD5. Higher levels of CD5 (peaked at day 7 after birth) were found in wild type and several types of mutant murine neonatal Tconv and Tregs when compared to their adult counterparts (35). However, the high affinity between TCRs and self-peptide/MHC complexes did not increase the likelihood to generate autoreactive T cells during neonatal period or incidence of autoimmune pathologies (36–38), at least in a rodent model with the transplantation of NOD thymi to NOD.*scid* mice (39). Instead, it promotes Tregs’ capability to undergo proliferation and likely, to modulate specific immune responses (40, 41). *Tdt*-deficient T cells also had an increased frequency for a given antigen, including self, commensal, and pathogenic ones (33, 34, 42). Such promiscuous peptide recognition is clearly an advantage to defend against a variety of environmental or infectious insults during neonatal period or during reconstitution after HSCT when the number of peripheral T cells is limited. Indeed, specific and competent CD8^+^ T cell responses against a range of viral infections (Vesicular Stomatitis Virus, Vaccinia Virus, and Lymphocytic Choriomeningitis Virus) *in vivo* have been observed in murine *Tdt*-deficient or neonatal T cells (34, 43, 44). In human samples, T cells in UCB had higher level of CD5 expression and higher precursor frequency for certain tumor-associated antigens or pathogens than T cells in adults (**Table 1**) (28, 42, 45). Together with delayed TdT expression and similar TCR sequencing feature between human fetal T cells and mouse neonatal T cells, it is believed, although more evidence is needed, that human TCR repertoire also has high cross-reactivity.

**Table 1:** Unique features of human Tconv and Treg cells in umbilical cord blood.

Human T cell types in UCB	Unique features (in comparison with adult counterparts)	Reference
**Tconv**	Higher CD5 expression in naïve CD4^+^ cells	(42)
	Higher frequency of pathogen-specific and PR1-specific clonotypes with smaller average clonotype size	(26, 45)
	Higher TCR diversity	(28, 46)
	Lower numbers of randomly added nucleotides in TCRs without affecting the functional diversity	(26)
	Higher percentage of nonfunctional TCR*β* mRNAs	(26)
	Higher numbers of public clones shared among samples	(26)
	More naïve CD4^+^ and CD8^+^ T cells	
	Upregulated Treg markers (*FOXP3* *TIGIT* and *IKZF2*),, after 14-day expansion	(28)
	Higher expression of inhibitory receptors including CTLA-4 (in CD4^+^CD28^+^ cells), LAIR-1, CD31, and CD200 in all T cells	(47, 48),
	Higher expression of costimulatory molecules including ICOS and CD26 in all T cells; higher/lower IFN-*γ* production and cytotoxicity upon stimulation *in vitro*	(49–51)
	Enhanced rejection of HLA-mismatched B cell lymphoma in a xenogeneic mouse model	(52)
	Transcriptional features associated more with cell cycle and innate immune responses and chromatin architecture of CD8^+^ T cells are similar to adult effector cells	(53, 54)
**Treg**	More diverse TCR repertoire	(28)
	Less effector-like cells	(28, 55)
	More clones with TCRs specific for autoantigens	(28)
	Higher integrin *β*7 expression and lower CLA expression	(55)
	Upon stimulation, Treg cells are more proliferative, have higher percentage of activated/effector cells, and perform better in the suppression assay	(27, 56–58)

## Thymic Selection in Early Life

During thymocyte development, the stochastic V(D)J recombination of TCR *α* and *β* chains inevitably generates thymocyte clones with high potential for self-reactivity. These autoreactive clones will either be removed by negative selection or develop into self-reactive thymic Tregs (tTregs) by agonist selection (59, 60). Thymic epithelial cells in the medulla (mTECs) are essential in these thymic selections by displaying a broad spectrum of self-peptide called tissue-specific self antigens (TSAs) to developing T cells (61). The expression of these TSAs in mTECs is regulated, in a significant part, by the transcriptional modulator autoimmune regulator (AIRE). Other regulators include but not limited to the transcription factor forebrain embryonic zinc fingerlike protein 2 (Fezf2) and mTECs’ autophagy machinery (62–64). Other cell types in the thymus, including cortical TECs, corneocyte-like mTECs (16), various types of dendritic cells (DC) (65–67), and B cells (68, 69), also contribute to negative selection of conventional T (Tconv) cells and agonist selection of tTregs. These different types of antigen presenting cells (APCs), with their different ways to sample and process self antigens, likely have non-redundant roles in thymic selection and in the determination of negative selection *versus* agonist selection (70, 71).

The uniqueness of thymic selection during neonatal period is not fully understood yet. Most of the evidence so far comes from murine studies. For instance, the interaction of developing thymocytes with medullary APCs may be limited due to small “islands” of thymic medulla in newborn animals in comparison with large and organized structure in adult ones (39). The spectrum of peptide presented by various thymic APCs is also different between neonatal and adult mice. Perinatal mTECs had a much lower ratio of HLA-DO : HLA-DM (non-classical MHC-II molecules that regulate peptide loading of MHC-II) and lower level of CD74/CLIP (MHC-II-associated invariant chain peptide) expression when compared to adult mTECs, indicating that mTECs in young animals have higher efficiency in loading a diverse repertoires of TSA peptides in the antigen-binding grooves of MHC-II molecules (27). MHC-II^hi^CD8*α* ^+^ conventional DC (cDC) that can cross-present diverse TSAs to thymocytes, however, are less in perinatal than in adult thymi (27). The seeding of migratory DCs, including B220^+^ plasmacytoid DCs and Sirpα^+^CD11b^+^ cDCs, to induce negative selection against peripheral self- and non-self antigens in the thymus also takes time, in particular when the number of DCs and the expression levels of MHC-II, CD86, and IL-12p70 in DCs were low during neonatal period (72–75).

The impact of the unique antigen presentation in neonatal thymus was demonstrated recently. Tconv cells specific for islet *β* cells can be observed within 1 week after birth, and the appearance of Tconv and tTreg specific for Peptidyl arginine deiminase, type IV (Padi4) and Adducin 2 (Add2) was restricted to 1–3-week-old mice (39, 76). Beyond the above indicated period, *β* cell-, Padi4- or Add2-reactive CD4 single positive T cells or tTreg cells were depleted in the thymus. The coincidence of bone marrow (BM)-derived cells accumulating in the thymus beyond weaning age indicates the likelihood of migratory DCs in inducing a late stage negative selection of these autoreactive T cells (76). The second piece of evidence comes from Aire-related studies. Mathis’s group found that the level of Aire expression and the repertories of Aire-dependent transcripts in mTECs were indistinguishable between <3-day-old and 5-week-old mice (27). However, thymectomy at day 3 after birth, turning off Aire expression before or shortly after birth, or tuning on Aire expression only after birth in the inducible *Aire* transgenic mice quickly led to the wasting disease and multiorgan autoimmune pathology (77), while turning off Aire expression beyond weaning age induced a different spectrum of pathologies (77–80). In addition, the multiorgan pathology in *Aire*-deficient mice could be ameliorated by the adoptive transfer of perinatal Tregs, but not adult Tregs (27). Collectively, these murine studies clearly demonstrate the differences in the antigen presentation machineries and post-selected repertoires between neonatal and adult thymi. Whether different selection machineries also exist in human thymi over the course of a lifespan is not clear. But infants who receive fully allogeneic thymi from unrelated infants generate Treg cells with diverse repertoires and Tconv being tolerant to self as well as the thymic transplant (81–83).

## Treg Cells in Early Life

Treg cells are an essential mode of immune tolerance that can be transferred into naïve animals to prevent rejection of tissue/cell transplantation, development of autoimmune diseases and atopic disorders, such as allergies (11–13, 84–86). The importance of Treg cells specifically in fetal tolerance is realized by the onset of IPEX (immune dysregulation, polyendocrinopathy, entheropathy, X-linked)-related autoimmunity at second-trimester in humans that lack functional FOXP3 (87). Using a *Foxp3-DTR* transgenic mouse system, we and Yang et al. showed that Treg depletion during the day 0–10 or day 7–11 age-window quickly resulted in significant weight loss and autoimmune pathology (27, 41). When Treg cells were depleted beyond weaning age (35–45-day window), only scattered individual mouse developed mild autoimmune inflammation (27). Collectively, these data demonstrate an active and tight control of fetal/neonatal autoimmune responses by Treg cells

In addition to self antigens, Treg cell-mediated immune tolerance to commensal microbiota-derived antigens is also critical at barrier sites. Notably, the preferential barrier sites for neonatal Treg regulation are the intestine in humans but the skin in mouse. In humans, Treg cells with gut tropism (integrin *β*7 expression) and resting phenotype are found most abundant at birth and decreased with age, while the frequency of Treg cells with skin tropism (cutaneous lymphocyte antigen (CLA) expression) and activated phenotype is increased later in life (55) (**Table 1**). IL-2 and IL-7, but not retinoic acid, promote the expression of *β*7 in Treg cells after thymic egress (55). Reduced tTreg cells in UCB were found to be associated with higher susceptibility to food allergies in infants (88). Thus, human neonatal tTreg cells may preferentially migrate to the gut and promote the establishment of mucosal immune tolerance (oral tolerance), in preparing for progressive exposure of microbial, diet, and environmental antigens after birth (89, 90). The reason for the delayed acquisition of skin homing potential in human neonatal Treg cells is not clear. But with impaired barrier function, such as in atopic dermatitis, late coming Tregs may increase the susceptibility to allergen sensitization through the skin (55).

In mouse, however, a unique neonatal Treg population was recently found to migrate to hair follicles and get activated at 1–2 weeks after birth, coinciding with the initial colonization of microbes to the skin (91, 92). Such rapid recruitment of Treg cells in neonatal skin depends on Ccl20–Ccr6 pathway stimulated by commensal bacteria and their surface molecules. Blocking Treg cell entry into hair follicles during neonatal window or colonization of bacteria during adult period all leads to increased antigen-specific effector T cells in the draining lymph nodes, demonstrating the importance of murine neonatal Tregs in promoting immune tolerance to skin commensal microbiota. It further indicates that certain chronic tissue inflammation in adults may be closely associated with impaired tolerance to commensal microbiota established during the neonatal period. Whether murine Treg cells (93–96) accumulate in other barrier sites, including lung and gut, during a defined early developmental period is not as clearly studied as the ones in the mouse skin.

A second difference between human and murine Treg cells is the timing of appearance, with the former emerging at gestational week 13 (97, 98) while the latter being detected in the thymus 2–3 days after birth (27, 99, 100). The frequency of human Treg cells in CD4^+^ T cells significantly increases during the second trimester then decreases during the third trimester. Within the first week after birth, Treg cell ratio rapidly increases again (56, 101, 102). Depletion of CD25^+^ Treg cells enhanced fetal T cell activation against self and maternal cells, but not against unrelated donor cells (103). Loss of FOXP3 leads to the occurrence of autoimmune inflammation specifically at second-trimester. Thus, the early appearance of human Treg cells in fetus plays a unique but critical role in maintaining self-tolerance as well as feto-maternal tolerance (8, 103, 104).

Murine neonatal Tregs and human fetal Tregs also have common features. They are more proliferative, have higher percentage of activated/effector cells, and perform better in the suppression assay *in vitro* when compared to adult Treg cells (27, 56). The transcriptome of human neonatal/fetal Tregs is also different from that of adult Treg cells, supporting the enhanced cell division and suppressive functions (57, 58).

## Origin of T Cells in Early Life

Although having different dynamics in T cell emergence, the origin of human and murine prenatal/perinatal T cells with distinct intrinsic properties, including short TCR, promiscuous antigen recognition, and high CD5 expression, is the same, *i.e.* both are derived from hematopoietic stem cells (HSCs) from fetal liver (53, 58, 105–108). High expression of *Lin28b* and high expression of let-7 microRNA mark the difference between fetal liver/thymus and adult BM/thymus, respectively. The detailed *in vivo* experiments in murine system further demonstrate that ectopic expression of *Lin28b* or loss of *Ezh2* in adult BM hematopoietic stem/progenitor cells (HSPCs) induces activation of fetal-specific genes (including let-7 target genes) in HSPCs and fetal-like lymphopoiesis, including the development of B-1 cells, marginal zone B cells, and *γδ* T cells (106, 109).

Both human or mouse fetal/neonatal CD4^+^ T cells preferentially differentiate into induced Tregs (iTregs) when compared to adult CD4^+^ T cells (58, 103, 110, 111). Inhibiting *Lin28b* in human fetal CD4^+^ T cells leads to let-7 upregulation and reduced Treg cell differentiation (112). Human fetal naïve T cells also express higher level of Helios, and deletion of Helios results in impaired Treg differentiation and regulatory function (113). These results demonstrate that fetal liver-derived T cells have unique intrinsic properties to promote Treg cell differentiation.

## Persistence of Neonatal T Cells in Adulthood

The uniqueness of neonatal T cells and their roles in immune tolerance are not restricted to early life. Using a fate-mapping model, Yang et al. found that the number and function of murine neonatal Tregs were stably maintained in adulthood (27). Thus, the adoptive transfer of the persisting neonate-derived Treg cells from adult mice suppressed the progression of multi-organ autoimmune pathology in *Aire*-deficient mice. Similarly, human fetal Treg cells specific for maternal antigens can be found more than a decade later, right into the teenage year (103). Therefore, Treg cells produced during a specific ontogenic window in early life are unique and essential in maintaining self-tolerance in adulthood.

Notably, the persistence of fetal T cells in young adults is not limited to Treg cells. The analysis of deep sequencing data of human TCR repertoire recently reveals that large numbers of naïve T cell clones without N-region addition (fetal origin) are public clones and also persist for decades (114). A better understanding of the impact of these persisting fetal/neonatal T cells on self-tolerance and immune responses against pathogen/tumor in adults will thus be important and may bring benefits in the development of vaccine and therapeutics.

## Early-Life T Cell Tolerance and Umbilical Cord Blood Transplantation

Allogeneic HSCT from an HLA-matched related or unrelated donor has been more and more widely used to treat patients with malignant or non-malignant hematological disorders (115). The HSCs used in the transplantation can be derived from BM, peripheral blood, or UCB. Multiple comparisons between the transplantation of UCB and BM/peripheral blood HSCs have shown that UCB grafts are associated with lower incidence of GVHD, and in some cases such as patients with pre-transplant persistent minimal residual disease, better long-term outcomes (116). When CD34^+^ cells from a third-party HLA-haploidentical donor were transplanted together with unrelated UCB cells, an early haploidentical engraftment was frequently replaced by durable UCB engraftment (117, 118). The distinct features of fetal liver-derived HSCs and Tconv/Treg cells described above may build the basis for these advantages in UCB transplantation (UCBT). Whether T cells reconstituted from UCBT could provide further benefits, such as better self-tolerance and lower incidence of autoimmune diseases later in life, will be an interesting question to investigate.

## Concluding Remarks

T cell-mediated immune tolerance is essential in preventing unwanted or self-reactive immune responses throughout life. The distinct features of prenatal/neonatal Tconv and Treg cells provide a unique layer of tolerance against maternal and self antigens, certain allergens, and commensal microbes-derived products. The in-depth investigation of these T cell populations in early life may shed light on a better understanding of the immune responses in infants, the early-life root of certain adult immune alterations, and the choice and prognosis of UCBT in treating hematological disorders.

## Author Contributions

LY and QG wrote the manuscript. DL and RJ gave critical comments and revision. All authors contributed to the article and approved the submitted version.

## Funding

This work was supported by grants from the National Key Research and Development Program of China (2017YFA0104500), the National Natural Science Foundation of China (32070897, 81471525, 31671244, 31872734), the Foundation for Innovative Research Groups of the National Natural Science Foundation of China (81621001), Beijing Natural Science Foundation (7202079), the Non-Profit Central Research Institute Fund of Chinese Academy of Medical Sciences, 2019PT320006.

## Conflict of Interest

The authors declare that the research was conducted in the absence of any commercial or financial relationships that could be construed as a potential conflict of interest.
